# Ventriculomegaly and cerebellar hypoplasia in a neonate with interstitial 11q 24 deletion in Jacobsen syndrome region

**DOI:** 10.1002/ccr3.1560

**Published:** 2018-05-22

**Authors:** Surasak Puvabanditsin, Charlotte Wang Chen, Marissa Botwinick, Karen Hussein, Joseph Mariduena, Rajeev Mehta

**Affiliations:** ^1^ Department of Pediatrics Rutgers Robert Wood Johnson Medical School New Brunswick New Jersey

**Keywords:** chromosomal anomaly, Jacobsen syndrome, Partial deletion 11q

## Abstract

Jacobsen syndrome (JS) is a rare contiguous gene disorder caused by partial deletion of the distal part of the long arm of chromosome 11 ranging in size from 7 to 20 Mb. We report a term male neonate with an interstitial deletion of about 12.3 megabase (Mb) of chromosome 11q24.1qter. Our case is the first reported newborn patient with 11q24 deletion.

## Introduction

Jacobsen syndrome (JS) is due to a contiguous gene deletion in the distal chromosome 11q. The deletion generally varies in size from 7 to 20 Mb, usually extends to the telomere, and the breakpoints occur within or distal to sub‐band 11q23.3. In a few cases, the break point may be at the FRA11B fragile site 1. Approximately, 85% of patients with JS have a de novo deletion, whereas 15% of cases it may be due to an unbalanced segregation of a familial balanced translocation or from other chromosomal rearrangements 2.

More than 200 cases of JS have already been reported with an estimated prevalence of 1/100,000 births and a female to male ratio of 2:1 2. The most common clinical features include prenatal and postnatal physical growth retardation, psychomotor retardation, and characteristic facial dysmorphism (skull deformities, hypertelorism, ptosis, coloboma, downslanting palpebral fissures, epicanthal folds, broad nasal bridge, short nose, v‐shaped mouth, and ears that are small, low‐set and posteriorly rotated). Abnormal platelet function, thrombocytopenia, or pancytopenia is usually present at birth. Patients commonly have malformations of the heart, kidney, gastrointestinal tract, genitalia, central nervous system, and skeleton. Ocular, hearing, immunological, and hormonal problems may also be present.

Newborns with Jacobsen syndrome may have difficulties in feeding and tube feeding may be necessary. Special attention should be devoted due to hematological problems. About 20% of children die during the first 2 years of life, most commonly related to complications from congenital heart disease, and less commonly from bleeding 2. For patients who survive the neonatal period and infancy, the life expectancy remains unknown.

## Clinical Report

A 2715‐g Mexican male neonate was born at 39 weeks’ gestation to a 31‐year‐old gravida (G) 4 para (P) 3 mother by vaginal delivery. Apgar scores were 7 and 8 at 1 and 5 min, respectively. Pregnancy was complicated with elevated serum alpha‐fetoprotein at 21 weeks of gestation. Obstetric sonogram at 22 weeks’ gestation revealed bilateral ventriculomegaly, absent corpus callosum, a large ventricular septal defect, and short long bones. Genetic studies had not been performed during the pregnancy. The family history was negative for congenital anomalies, and there was no history of consanguinity or in utero exposure to known teratogens.

**Figure 1:**
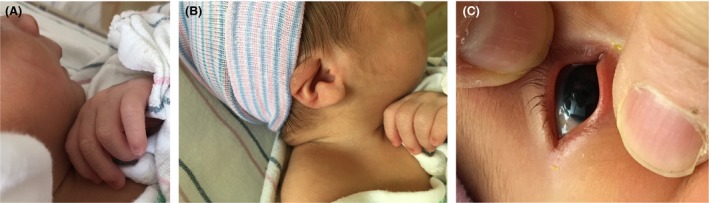
(A) Note micrognathia, thin and slender fingers. (B) Note low‐set and dysplastic ear. (C) Note megalocornea.

**Figure 2:**
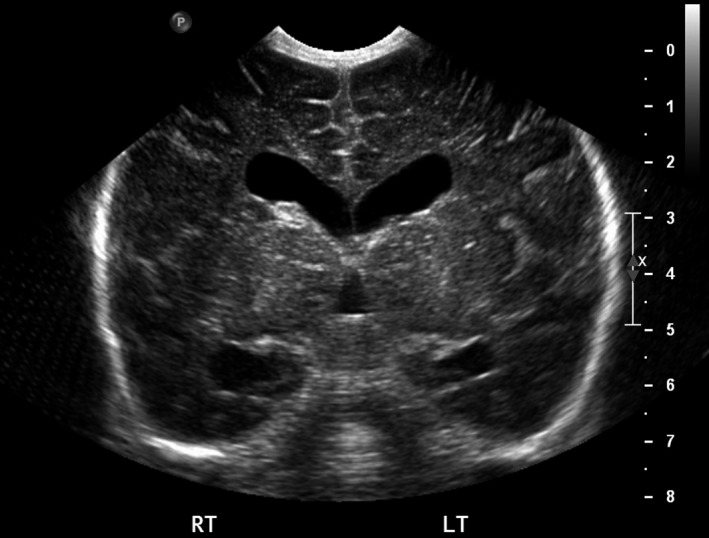
Neurosonogram showed right subependymal hemorrhage and dilation of lateral ventricles.

**Figure 3:**
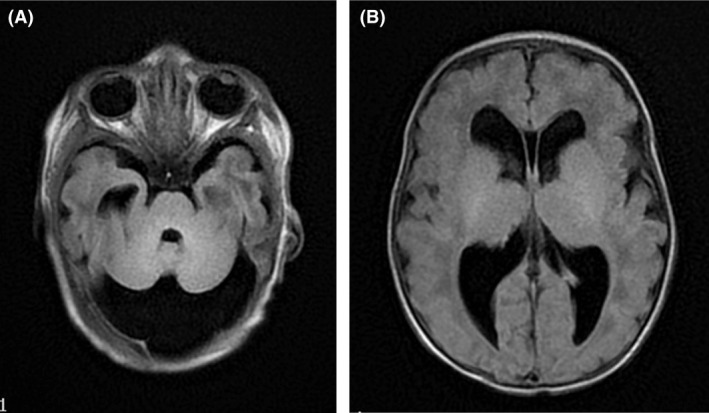
(A) Cranial MRI shows cerebellar hypoplasia. (B) Cranial MRI shows bilateral ventriculomegaly.

Physical examination revealed a weight of 2715 g (15th centile), length of 51 cm (60th centile), and head circumference of 35 cm (50th centile). Multiple anomalies were noted at birth including short neck, low‐set and dysplastic ears, large fontanels, hypertelorism, flat midface, megalocornea, high and narrow palate, long philtrum, micrognathia, thin and slender fingers, and supernumerary nipple (Fig. 1). Persistent thrombocytopenia was noted right after birth and throughout the hospitalization. The range of platelet count was 43,000–68,000/mm^3^. Neurosonogram showed right germinal matrix hemorrhage (Fig. 2). MRI of the brain revealed bilateral ventriculomegaly and residuals of germinal matrix hemorrhage, enlarged cisterna magna, and hypoplasia of cerebellar vermis (Fig. 3). Echocardiography showed a large perimembranous ventricular septal defect (VSD) with posterior extension and extensive aneurysmal tissue. There was no coarctation of the aorta. The infant remained in the hospital for 21 days because of feeding difficulties requiring gavage feeding and tachypnea secondary to mild congestive heart failure. His platelets remain low (<75,000/mL) but stable and he has experienced no significant bleeding episodes.

## Cytogenetic and Molecular Studies

**Figure 4:**
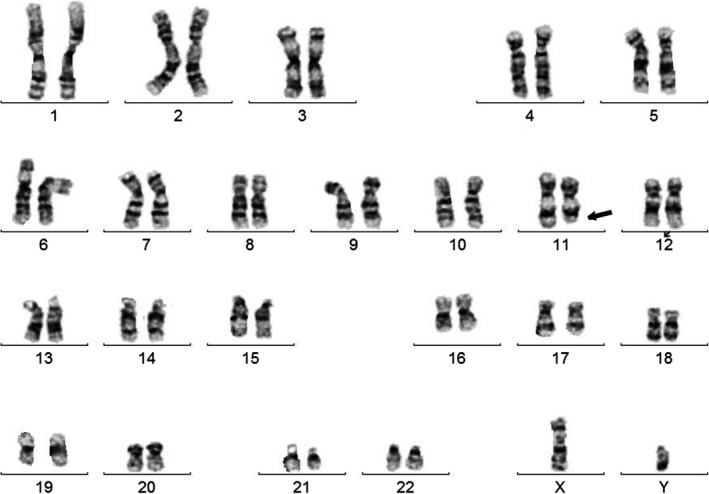
Karyotype shows terminal deletion of the long arm of chromosome 11 [46XY,del (11) (q23q25)] (arrow).

**Figure 5:**
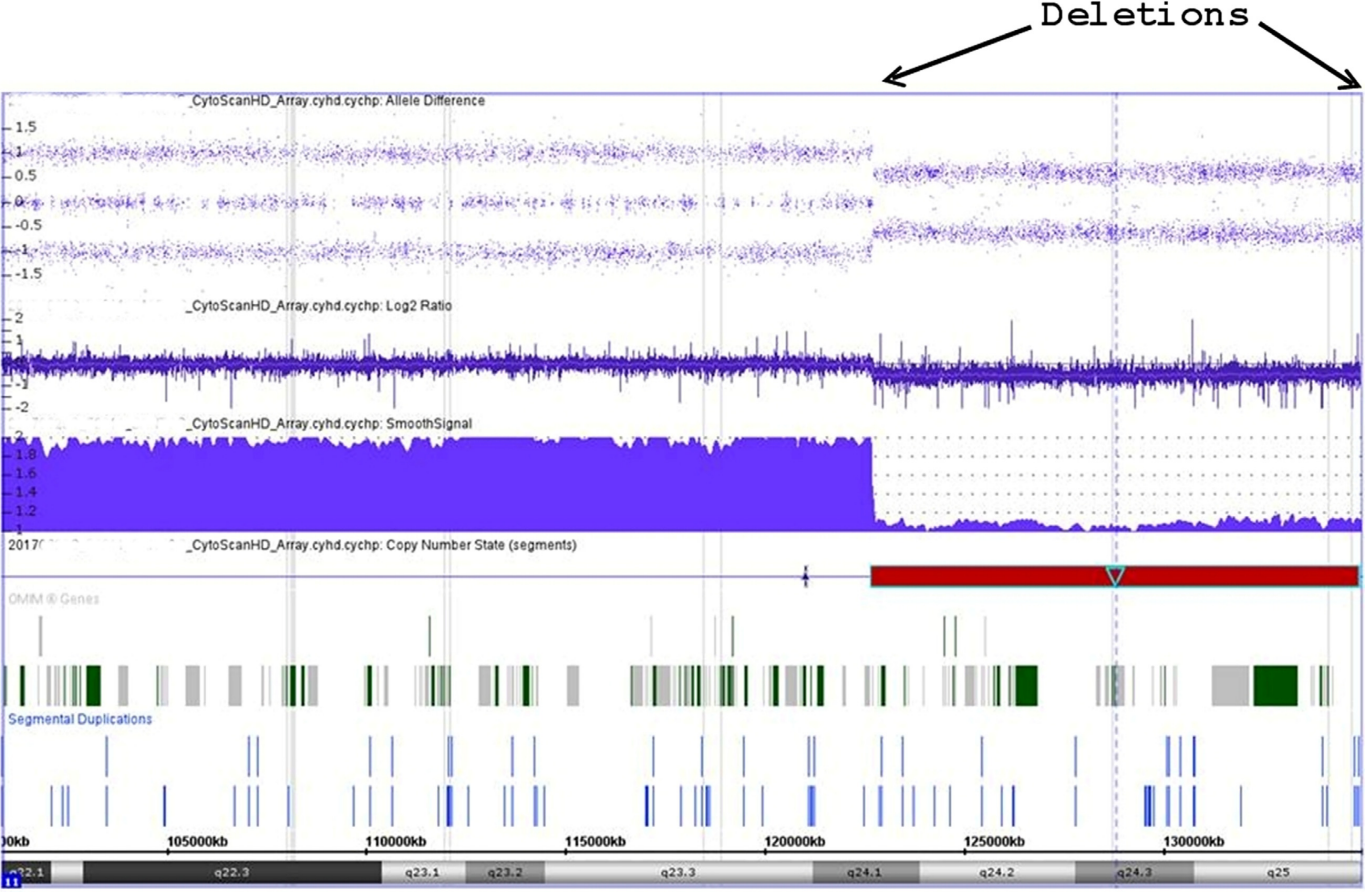
aCGH, at a resolution of about 0.88 Kb, shows an interstitial deletion of chromosome region 11q24.1 to qter (arrows).

Chromosome analysis showed a deletion of the long arm of chromosome 11 –46,XY,del (11) (q23q25) (Fig. 4). The whole genome SNP (Single Nucleotide Polymorphisms) microarray analysis was performed using the Affymetrix CytoScan HD platform which uses over 743,000 SNP probes and 1,953,000 NPCN probes. There was a 12.3 megabase (Mb) deletion between 11q24.1 and qter – arr [bg19] 11q24.1.1q25 (122,692,285–134,938,470) × 1 (Fig. 5). SNP oligonucleotide microarray analysis (SOMA) indicated a loss of chromosome 11 from positions 122,692,285–134,938,470. The deletion interval contains several numerous OMIM genes [proximal OMIM gene: CRTAM] consistent with distal 11q deletion‐Jacobsen syndrome. In addition, there was a high density of short runs (1–8 Mb) of allele homozygosity (ROH) observed throughout genome, consistent with a limited gene pool present in isolate populations. The finding reflects an increased risk of recessive allele pairing present in that population.

## Discussion

Interstitial deletion at band 11q24 is very rare. To our knowledge, only seven patients with interstitial deletions at 11q24 (identified by array CGH) have been reported, and none were diagnosed in the newborn period 2, 3, 4, 5, 6, 7, 8. Our case revealed distinct phenotypic findings of a neonate with 11q24 deletion. There are considerable variations in the phenotypic spectrum and it correlates with the size of the deletion. Recent advances in molecular cytogenetic techniques, for example, microarray analysis have further delineated the genes responsible for the different phenotypes. The clinical and genetic findings of the seven reported patients, including our case, are shown in Table 1.

**Table 1:** Clinical features of JS patients with interstitial deletion 11q24

	Our case	Tassano et al. 2016	Muruani et al. 2015	So et al. 2014	Guerin et al. 2012	Coldren et al. 2009 (14 cases)	Tyson et al. 2008 (2 cases)
Age	Newborn	19 months	21 years	67 years	4 years	Not reported	8 months
Growth retardation	No		Short stature				
Brain image	Ventriculomegaly, hypoplasia of the vermis			Periventricular nodular heteropia			
Face	Hypertelorism, micrognathia, high arch palate	Hypertelorism	High forehead, large nasal bridge	Mild dysmorphism	Hypertelorism,trigonocephaly		Prominent forehead, round face, broad nose
Ophthalmologic	Megalocornea	Small right eyelid with abduction defect		Small cataract	Deep set eyes, prominent suprsorbital ridge		
Ears/Hearing	Low‐set/dysplastic ears	NR	Low‐set ears				Mild sensorineural hearing loss, overfolding of the helices
Cardiac anomaly	Ventricular septal defect (VSD), atrial septal defect (secondum)	Mitral valve anomaly			VSD		
Hematologic	Thrombocytopenia	Thrombocytopenia, anemia			Thrombocytopenia		
Urinary	None	Mega ureter					
Extremities	Thin and slender fingers			Right limb reduction			Short 5th finger, squaring fingertips
Psychomotor	N/A	Global developmental delay	Moderate developmental delay, autism	Seizure	Developmental delay, autism	Severe developmental delay	Developmental delay
Cytogenetic	del (11) (q23q25)	del (11) (q24.2q24.3)		del (11) (q24.2q24.3)	del (11) (q24.2q24.3)		del (11) (q24.2q24.3)
Array CGH	Chr11:12,692,285‐134,938,470 (Hg 19) affirmetrix 743K SNP Platform (average resolution ~ 0.88 kb)	Chr11:127,217,775‐129, 666,990 (Hg 19) Human Genome CGH Microarray Kit G3 180 (average resolution ∼13 Kb) (Agilent Technologies)	Chr11:126,633,940‐132, 060,375 (Hg 18) Illumina Human 1 M(illumine, Inc, San Diego, California)	Chr11:125,780,310–128, 942,331 (Hg 19) Custom designed 4 × 180K oligonucleotide microarray (Oxford Gene Technology (OGT).	Chr11:126,767,670‐129, 667,131 (Hg 18) 4 × 44 K microarray platform (average resolution ∼75 Kb) (Agilent Technologies)	Chr11:120,476,074‐128, 148,010 (Hg 18) Affymetrix 500 K SNP Platform (average resolution ∼10 kb) or Agilent 44B platform (average resolution ∼75 kb), (Agilent Technologies	Chr11:124.29–129.03 (Hg 17) Spectral Genomics 1 Mb array (Spectral Genomics, Houston, TX)
Mb	12.3	2.45	5.4	3.16	2.9	7.67	4.74
Origin		Human Genome CGH Microarray Kit G3 180 (average resolution ∼13 Kb) (Agilent Technologies)					De novo

The size of the imbalance ranged from 2.45 Mb to 7.67 Mb, and the deletion was de novo in one patient. Only one patient showed growth retardation. Thrombocytopenia and cardiac problems were observed in three of the eight cases, craniofacial malformations were present in six, hearing, and urinary abnormalities were noted in one. Three patients showed ophthalmologic anomalies. All presented with neurological problems including developmental and language delay, hypotonia, seizures, and an autism spectrum disorder. Our case represents a newborn with new phenotypic findings: megalocornea, bilateral ventriculomegaly of lateral ventricles, enlarge cisterna magna, and cerebellar vermis hypoplasia. Because we do not have the required information on the mother, we cannot say that our case de novo. The 11q24qter region deletion present in our patient contains about 174 genes including BSX, NRGN, ETS‐1, FLI‐1, and RICS (ARHGAP32) which are implicated in causing JS 1, 8, 9.

In our patient, a cranial ultrasound evaluation soon after birth revealed a germinal matrix hemorrhage. The majority (90%) of cases with 11q terminal deletion have the Paris‐Trousseau syndrome (PTS), a defect in platelet development characterized by neonatal thrombocytopenia and persistent platelet dysfunction 6, 10. The platelets of PTS patients are larger than normal, contain giant granules, and exhibit abnormal platelet adhesion. This platelet defect is associated with *ETS‐1, FLI‐1, NFRKB,* and JAM3 genes 8. The *FLI‐1* gene, which maps to chromosome sub‐band 11q24, is a proto‐oncogene from to the *ETS* (erythroblast transformation‐specific) family of transcription factors. In‐vivo and in‐vitro studies have shown that FLI‐1 plays a crucial role in the differentiation of megakaryocytes and interacts with the various genes involved in vasculogenesis, hematopoiesis, and intercellular adhesion. Heterozygous loss of the *FLI‐1* gene is associated with dysmegakaryocytopoiesis 1, 11.

Over 50% of the cases have abnormal brain imaging, some kind of structural abnormality of the brain, including: enlarged ventricles, cerebral atrophy, and agenesis of corpus callosum or pachygyria 2, 8. Candidate genes include FEZ1, predominantly expressed in the brain and involved in axonal outgrowth, and RICS, expressed during neural development and which may regulate dendritic spine morphology and strength 8. Exploration of brain structures using MRI in one reported case (an adult) revealed a decrease in the gray matter volume, especially noticeable in the occipital region (approximately 2 SD) 3. It is difficult to find the gene(s) associated with cognitive impairment in patients with JS due to the relatively high number of compelling candidate genes located at 11q24. So far, no gene has been formally identified as the cause of the cognitive phenotype of patients with JS. An evolutionarily highly conserved homeobox gene called *BSX* and mapped in 122.3 Mb is expressed during early brain development and has been proposed as a candidate gene for global cognitive development 12. The gene Beta‐1–3‐glycoronyltransferase (B3GAT1), located in 11q25 at 133.77 Mb, is expressed in the brain. The genes NRGN and KIRREL3 are involved in synaptic plasticity, and *ARHGAP32, NTM, OPCML* involved in axon guidance and outgrowth 5, 7. Impaired synaptic plasticity and learning disabilities have been reported in knockout mice 1, 13. Abnormalities of the white matter appear to map between 124.6 and 129.03 Mb. Hypothetical candidate genes include *FEZ1*, involved in axonal outgrowth, and *RICS*, which is highly expressed during neural development 1, 8. They may regulate dendritic spine morphology and strength 14.

More than 50% of patients with JS have congenital heart defects 10. Of these, two‐thirds are ventricular septal defects or left sided heart lesions. The most severe form being the hypoplastic left heart. Candidate genes that have been proposed to contribute to these features include JAM3 and *ADAMTS8*. JAM3 is expressed in the heart and peripheral nerves, and is suggested to play a role in congenital heart anomalies 15. However, deletion of JAM3 in mice failed to yield cardiac abnormalities, and terminal deletions that do not involve *JAM3* have been seen in individuals with cardiac anomalies, suggesting another gene or perhaps multigenic mechanisms 16, 17. *ADAMTS8* is important in angiogenesis and is considered a candidate gene for the congenital heart defect 8. However, in our patient who has a VSD, neither of these genes was found deleted.

A candidate gene found deleted in our patient was *ETS‐1*, which in mice is thought to play a role in cardiac development 17. Previous deletion mapping has identified *BARX2* as a candidate gene for the craniofacial features (facial dysmorphism and/or craniosynostosis) reported in JS patients 1, 8, 18, 19, 20. Patients who did not have a deletion of this gene did not have the facial phenotype seen in JS 8. The craniofacial anomalies seen in our patient, who had a deletion of the *BARX2* gene, support the assumption that its deletion is a contributory factor for the dysmorphic features observed. *NCAM* and *Mfrp* are candidate genes in this region that may be responsible in ocular manifestations of JS 21.

A number of tumor‐related genes that have been mapped to the 11q distal region include *EST1, CHK1, BARX2, OPCML, FLI‐1* 1 *TECTA*, a gene coding for the noncollagenous component of the tectorial membrane of the inner ear, may be involved in neurosensorial deafness. The *KCNJ1* and *ADAMTS15* genes, which are expressed in the fetal liver and kidney, may be related to kidney malformations 1, 4, 8.

Tassano et al. (2016) and Guerin et al. (2012)) reported two patients with a very similar deletion sizes but their cases showed phenotypic discrepancies. The variable penetrance observed in the phenotypic spectrum of JS patients suggests that there are other factors involved that could modulate the severity of the phenotype 4. Grossfeld et al. 10 and Penny et al. 18 had defined critical regions defined based on conventional karyotypes and a selection of DNA markers from the 11q terminal region. Considering the differing phenotypes of JS, deletion sizes, and critical regions defined by a particular phenotype, it could be due to monosomy of single genes, or cumulative effect of a combination of contiguous genes, or perhaps due to gene–gene interaction. The use of whole genome array‐CGH in JS patients should facilitate a more accurate assessment of the types and sizes of chromosomal rearrangements.

In summary, we report a neonatal case of Jacobsen syndrome associated with the 11q24.1qter deletion. The megalocornea, neurosonogam, and MRI images have not previously been described in an infant with JS. Our report could help delineate phenotypic findings of JS in a newborn, and clinicians should be aware of the possibility of intracranial hemorrhage in Jacobsen Syndrome neonates with PTS.

## Authorship

The authorship list and author's contribution. SP: corresponding author, drafting/preparing/final approving the manuscript. CWC: was responsible to acquire the data and revise the manuscript. MB: was responsible to acquire the data and revise the manuscript. KH: was responsible to acquire the data and revise the manuscript. JM: was responsible to acquire the data and revise the manuscript. RM: was responsible to analyze/revise/approve the manuscript.

## Conflict of Interest

None declared.
